# Transcriptome Profiling of Sugarcane Roots in Response to Low Potassium Stress

**DOI:** 10.1371/journal.pone.0126306

**Published:** 2015-05-08

**Authors:** Qiaoying Zeng, Qiuping Ling, Lina Fan, Yu Li, Fei Hu, Jianwen Chen, Zhenrui Huang, Haihua Deng, Qiwei Li, Yongwen Qi

**Affiliations:** Guangdong Key Lab of Sugarcane Improvement & Biorefinery, Guangzhou Sugarcane Industry Research Institute, Guangzhou, China; University of Delhi South Campus, INDIA

## Abstract

Sugarcane is the most important crop for supplying sugar. Due to its high biomass, sugarcane needs to absorb a large amount of potassium (K) throughout its lifecycle. In South China, a deficiency of K available in soil restricts the production of sugarcane. Increasing the tolerance of sugarcane to low-K will be an effective approach for improving survival of the crop in this area. However, there is little information regarding the mechanism of tolerance to low-K stress in sugarcane. In this study, a customized microarray was used to analyze the changes in the level of transcripts of sugarcane genes 8 h, 24 h and 72 h after exposure to low-K conditions. We identified a total of 4153 genes that were differentially expressed in at least one of the three time points. The number of genes responding to low-K stress at 72 h was almost 2-fold more than the numbers at 8 h and 24 h. Gene ontology (GO) analysis revealed that many genes involved in metabolic, developmental and biological regulatory processes displayed changes in the level of transcripts in response to low-K stress. Additionally, we detected differential expression of transcription factors, transporters, kinases, oxidative stress-related genes and genes in Ca+ and ethylene signaling pathways; these proteins might play crucial roles in improving the tolerance of sugarcane to low-K stress. The results of this study will help to better understand the molecular mechanisms of sugarcane tolerance to low-K.

## Introduction

Potassium (K) is one of the most important macronutrients for plants, playing vital functions in maintaining plasma membrane potential, ion homeostasis, enzyme activation, signal transduction, and many other physiological processes [1]. There are four forms of K in the soil: soluble K, exchangeable K, fixed K, and lattice K. However, plants can only take up soluble K from soil solutions. Generally, the concentration of K^+^ in soil solutions varies widely (from 0.1 to 6.0 mmol L^-1^) [2]. In many areas, the soil K^+^ concentration is extremely low, usually less than 0.3 mmol L^-1^ [3], and the growth of plants is inhibited by this deficiency. However, plants can initiate a series of physiological and molecular reactions to improve their tolerance to K deficiency. In recent years, a great deal of research has focused on the molecular mechanisms of K uptake, loading and transport in plants, and has demonstrated that genes in both high- and low-affinity K transport systems may be involved in K acquisition and homeostasis in plants under low-K conditions [2,4]. Many genes involved in high-affinity K transport, such as *AtKUP3* [5], *AtHAK5* [6,7], *HvHAK1* [8] and *OsHAK1* [9], are induced by low-K stress conditions, and K deficiency triggers the expression of K channel genes such as *TaAKT1* in wheat [10]. In addition, genes involved in K signal transduction, such as calcium signaling genes (CBL-CIPK complexes) [11], ethylene signaling genes [12] and transcription factors [13], are known to play important roles in improving the tolerance of plants to low-K. Transcriptomic analysis of the response of *Arabidopsis*, rice, soybean and wild barley to K deficiency has revealed that genes related to metabolism, ion transport, signal transduction and protein phosphorylation are altered in the level of transcripts [14–17].

Sugarcane (*Saccharum species* hybrid L.) is an important crop for the production of sugar. Due to its high biomass, it demands a large amount of nutrients for optimum productivity. Generally, 1.00–2.50 kg of K_2_O will be absorbed in the production of a ton of cane, and approximately 790 kg ha^-1^ potassium per year will be removed from soil by sugarcane [18,19]. Previous work has indicated that the number, height and sucrose content of millable stalks at harvest will decline under low-K conditions [20]. Indeed, the available K in soil is often deficient in most areas of South China, which is the primary region where sugarcane is cultivated. Enhancement of K uptake under low-K conditions will therefore benefit cane production. Genetically improving the uptake capacity of K from soil is an effective method to enhance the K use efficiency of plants [21]. However, few details are known regarding the molecular mechanisms underlying K absorption, transport and regulatory pathways related to K uptake in sugarcane under low-K stress conditions. Recently, high-throughput technologies have been widely used to analyze the gene expression patterns of plants under low nutrient stress [22,23]. Transcriptome profiling of rice, *Arabidopsis* and soybean in response to low-K stress has previously been initiated using microarray and transcriptome sequencing, and many genes that respond low-K stress have been identified. Knowledge about these genes will provide valuable information on the molecular mechanism of K absorption and transport under low-K stress [14,16,17]. Here, a customized array was used to detect changes in the level of transcripts of sugarcane genes under low-K conditions. Many genes encoding transporters, kinases and transcription factors were differentially expressed in response to low-K conditions. These results will provide clues regarding the molecular mechanisms by which sugarcane is able to cope with K deficiency.

## Materials and Methods

### Plant growth conditions and low-K-induced stress

All setts of sugarcane cultivar ROC22 were cut into single-bud setts and then sterilized with 5% carbendazim for 10 min. All single-bud setts were planted in quartz for germination. Seedlings with four leaves were hydroponically cultured in a greenhouse under natural lighting. The greenhouse was controlled to maintain a temperature of 30°C. The nutrient solution used was modified Magnavaca’s solution [24] containing 1.5 mM NH_4_NO_3_, 1 mM KCl, 1 mM CaCl_2_, 0.5 mM Mg(NO_3_)_2_, 0.155 mM MgCl_2_, 0.045 mM KH_2_PO_4_, 1.643 mM MgSO_4_, 11.8 μM MnCl_2_, 0.61 μM (NH_4_)_6_Mo_7_O_24_, 33 μM H_3_BO_3_, 3.06 μM ZnSO_4_, 0.8 μM CuSO_4_, 0.077 mM FeSO_4_, and 0.077 mM Na_2_ EDTA (pH = 6.0). Six seedlings were planted in 10 L plastic basins, and a total of nine basins were planted: three for measuring the K concentration in the plants and six for microarray and qRT-PCR analysis. The nutrient solution was replaced every week. After two weeks, all plants (with 8–10 adventitious roots) were transferred to a low-K nutrient solution containing 0.1 mM KCl.

### Determination of K content

The roots and shoots of three seedlings from three basins were separately collected at 0 h (Control), 8 h, 24 h, 72 h and 7 d after initiating low-K treatment. All plants were killed at 105°C and then dried in an oven at 75°C for 2 d. Dried shoots and roots were ground, and approximately 1-g samples were moist-ashed with H_2_SO_4_-H_2_O_2_. The concentration of K in each digest was measured using a flame photometer (Model 425, Sherwood Scientific Ltd, Cambridge, UK) [25]. The concentration of K in the roots and shoots was also calculated.

### RNA extraction and microarray hybridization

Three adventitious roots (approximately 10 cm from the root tip) from each of six seedlings in six independent basins were collected at 0 h (CK), 8 h, 24 h and 72 h after initiating low-K treatment. A total of 18 adventitious roots were collected at each time point and were mixed to create one sample. Samples from four time points were immediately frozen in liquid nitrogen and stored at -80°C until RNA extraction. Total RNA was extracted using TRIZOL Reagent (Life Technologies, Carlsbad, CA, US) following the manufacturer’s instructions, and RNA integrity number (RIN), a measure of RNA quality, was determined as using an Agilent Bioanalyzer 2100 (Agilent Technologies, Santa Clara, CA, US). Total RNA was further purified with the RNeasy mini kit and RNase-Free DNaseSet (QIAGEN, GmBH, Germany). A customized sugarcane genome array ordered from Agilent Technologies was used in this study. The array contained 61,637 probes, which were designed by Shanghai Biotechnology Corporation according to sequences derived from transcriptome sequencing of sugarcane subjected to low-K, drought, salt and pathogenic stress (our previous unpublished research), as well as sugarcane genes deposited in GenBank (S1 Table, GPL19240 deposited in http://www.ncbi.nlm.nih.gov/geo/query/acc.cgi?acc=GPL19240). Total RNA was amplified and labeled with the One-Color Low Input Quick Amp Labeling Kit (Agilent Technologies, Santa Clara, CA, US) according to the manufacturer’s instructions. Labeled cRNA was purified with the RNeasy Mini Kit (QIAGEN, GmBH, Germany). Each slide was hybridized with 1.65 μg of Cy3-labeled cRNA using the Gene Expression Hybridization Kit (Agilent Technologies, Santa Clara, CA, US) in a Hybridization Oven (Agilent Technologies, Santa Clara, CA, US), according to the manufacturer’s instructions. After 17 h of hybridization, the slides were washed in staining dishes (Thermo Shandon, Waltham, MA, US) with the Gene Expression Wash Buffer Kit (Agilent Technologies, Santa Clara, CA, US), according to the manufacturer’s instructions. The slides were scanned with an Agilent Microarray Scanner (Agilent Technologies, Santa Clara, CA, US) with the following default settings: dye channel = green; scan resolution = 3μm, 20 bit. Data were extracted with Feature Extraction software 10.7 (Agilent Technologies, Santa Clara, CA, US). Raw data were normalized with the Quantile algorithm using Gene Spring Software 11.0 (Agilent Technologies, Santa Clara, CA, US).

### Data processing and analysis

First data with low raw signals (less than 2^7^) in all four arrays were filtered. Pair-wise comparisons were performed to assess changes in gene expression of 8 h vs. 0 h, 24 h vs. 0 h and 72 h vs. 0 h. A 2-fold change was used as the threshold for determining differentially expressed genes. A BLAST search was performed on sequences of differentially expressed genes against the nr database in NCBI. GO analysis was carried out according the sequences used for customizing the microarray and the web set found at http://www.geneontology.org (P<0.05). MeV 4.6.0 was used for hierarchical cluster analysis of differentially expressed genes.

### Quantitative real-time (qRT)-PCR analysis

The remaining adventitious roots of six seedlings used for the microarray analysis at each time point were collected separately. Three of the six samples at each time points were used for qRT-PCR to validate the microarray results. RNA was extracted with the EasyPure Plant RNA Kit (TransGen Biotech, China). First-strand cDNA was then synthesized using the PrimeScript Reagent Kit with gDNA Eraser (TaKaRa, Japan) from 1 μg of total RNA in a 20-μl reaction volume. Fifteen differentially expressed genes with different functions were selected to validate the microarray results. Primer Premier 5.0 software (Lynnon Corporation) was used to design gene-specific primers (S2 Table). The qRT-PCR was performed using 0.5 μl of cDNA in a 25-μl reaction volume with SYBR Premix ExTaq (TOYOBO, Japan) on an ABIPRISM 7500 Sequence Detection System (Applied Biosystems). The qRT-PCR program was as follows: 95°C (10 s) followed by 45 cycles of 95°C (5 s), 60°C (30 s), and 72°C (30 s). The products were further analyzed by a dissociation curve program at 95°C (15 s) ramping down to 60°C (1 min) followed by 95°C (15 s). In each qRT-PCR experiment, each gene was analyzed in triplicate with different cDNAs synthesized from three biological replicates. The genes with unimodal dissociation curve were selected for subsequent analysis. Relative fold changes in gene expression were calculated using the comparative ΔΔCt method [26], and GAPDH was used as an endogenous reference gene. For microarray validation, the 2^-ΔΔCt^ values were calculated for each gene in each sample and log_2_ transformed.

### Statistical analysis

Significant differences in the K concentration in shoots or roots at 0 h, 8 h, 24 h, 72 h and 7 d after initiating low-K stress were examined using the IBM SPSS statistical software package (version 19), followed by Duncan’s Multiple Range Test (DMRT). Differences with P<0.05 were considered significant.

## Results

### Effect of the K concentration on sugarcane shoots and roots

**Fig 1:**
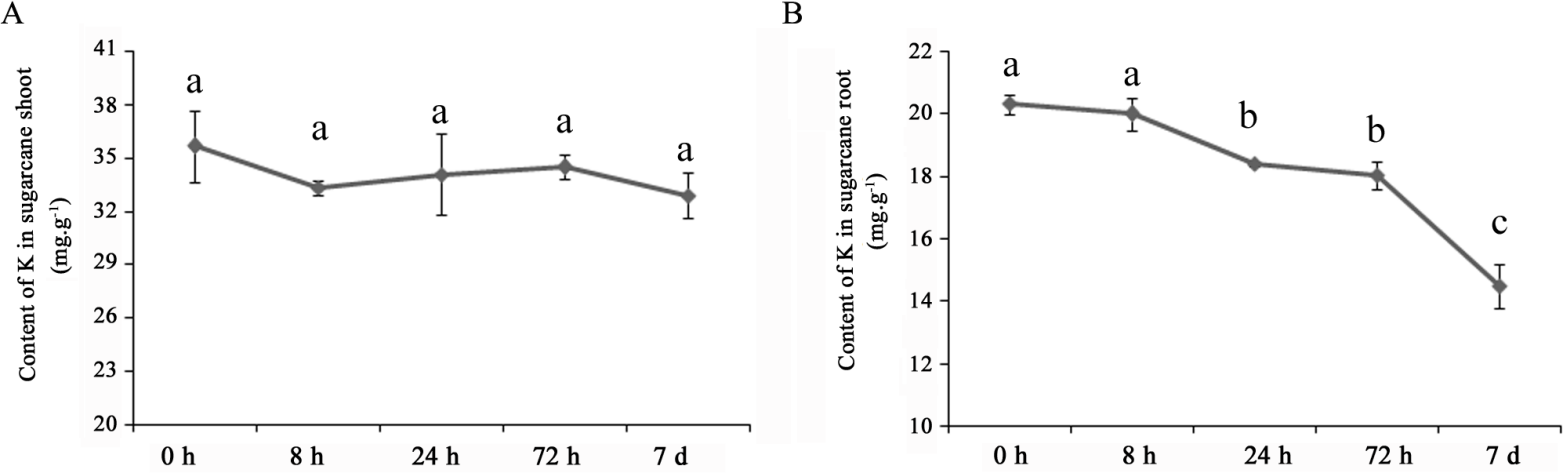
K concentration in sugarcane shoots and roots under low-K stress. The K concentration of sugarcane was measured after 0 h, 8 h, 24 h, 72 h and 7 d of low-K stress. A) The K concentration in shoots at 0 h, 8 h, 24 h,72 h and 7d; B) The K concentration in roots at 0 h, 8 h, 24 h,72 h and 7d. The different lowercase letters above the error bar (standard error) indicate significant differences (P<0.05) in the K concentration between 0 h and the other four time points (8 h, 24 h, 72 h and 7d); n = 3.

To investigate the effects of low-K stress on sugarcane, the K concentration in shoots and roots was measured 0 h, 8 h, 24 h, 72 h and 7 d after exposure to low-K conditions (Fig 1). The K content of sugarcane shoots decreased slightly after the initiation of low-K stress; however there were no differences between the 0 h time point and any of the other four time points (Fig 1A). Interestingly, a significant difference in the K concentration at five time points was detected in sugarcane roots (P<0.05). The K concentration in roots decreased rapidly after only 8 h of exposure to low-K stress. After that time point it decreased further, reaching statistical significance after 24 h (Fig 1B). These results revealed that the K concentration might decrease earlier in roots compared with shoots during K deficiency.

### Microarray analysis of genes that were differentially expressed under K deficiency

**Fig 2:**
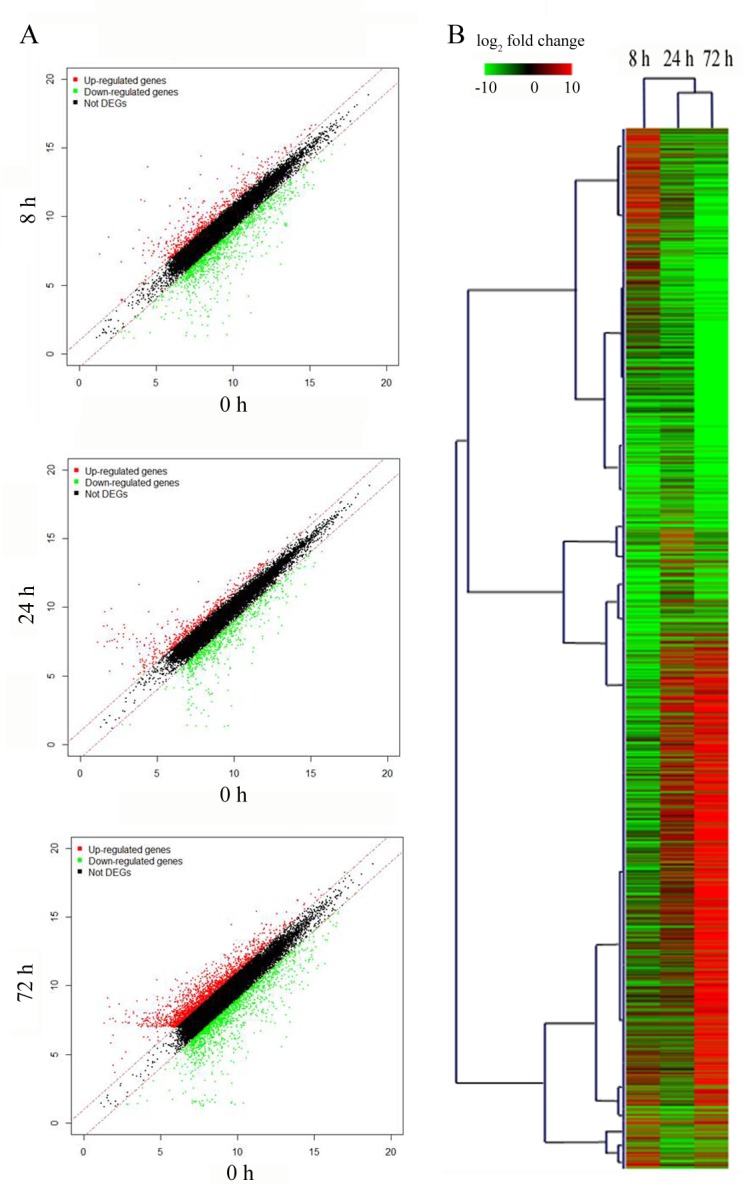
Gene expression detected by microarray after different periods of low-K stress. **A)** Scatter plots of the normalized signal intensities of approximately 21516 genes on the microarray. Log_2_ intensities for each spot on the microarray are plotted on the x and y axes with signals from root tips stressed for 0 h and 8 h, 0 h and 24, and 0 h and 72 h. The diagonal lines represent fold change cutoffs of 2. The red spots represent up-regulated genes and the green spots indicate down-regulated genes. **B)** Hierarchical cluster analysis of genes which were responsive to low-K stress. Genes are displayed using different colors, and relative expression levels are illustrated by a color gradient from low (green) to high (red).

To determine the global transcriptome responses to K deficiency in sugarcane, a microarray containing 61,637 primers was customized according to sequences derived from transcriptome sequencing of sugarcane subjected to low-K, drought, salt and pathogenic stress, as well as sequences of sugarcane genes deposited in GenBank. The cDNAs synthesized from root RNA derived from samples obtained 0, 8, 24 and 72 h after the initiation of low-K treatment were hybridized to the customized microarrays. Of the 61,637 spots on the array, 41.75% of the probes produced signals of up to 2^7^ in at least one of the four arrays and were used for further analysis. A scatter plot of normalized signal intensities from 8 h vs. 0 h, 24 h vs. 0 h and 72 h vs. 0 h revealed that the expression of the vast majority of transcripts remained unchanged (Fig 2A). A fold change cut-off value of 2.0 was used to identify genes that were responsive to low-K stress. A total of 1545 genes at 8 h, 1053 genes at 24 h and 3155 genes at 72 h were identified as being differentially expressed during low-K treatment (S3 Table and Fig 2). More genes responded to low-K stress at 72 h compared with 8 h and 24 h. Hierarchical cluster analysis showed that the expression pattern of genes at 24 h was similar to that at 72 h but different form that at 8 h (Fig 2B). This result suggested that genes that are differentially expressed at 8 h might be involved in short-term responses to K deficiency, while the long-term response to K deficiency was initiated after 24 h of exposure. All of the low-K-responsive genes are presented in S4–S9 Tables.

**Fig 3:**
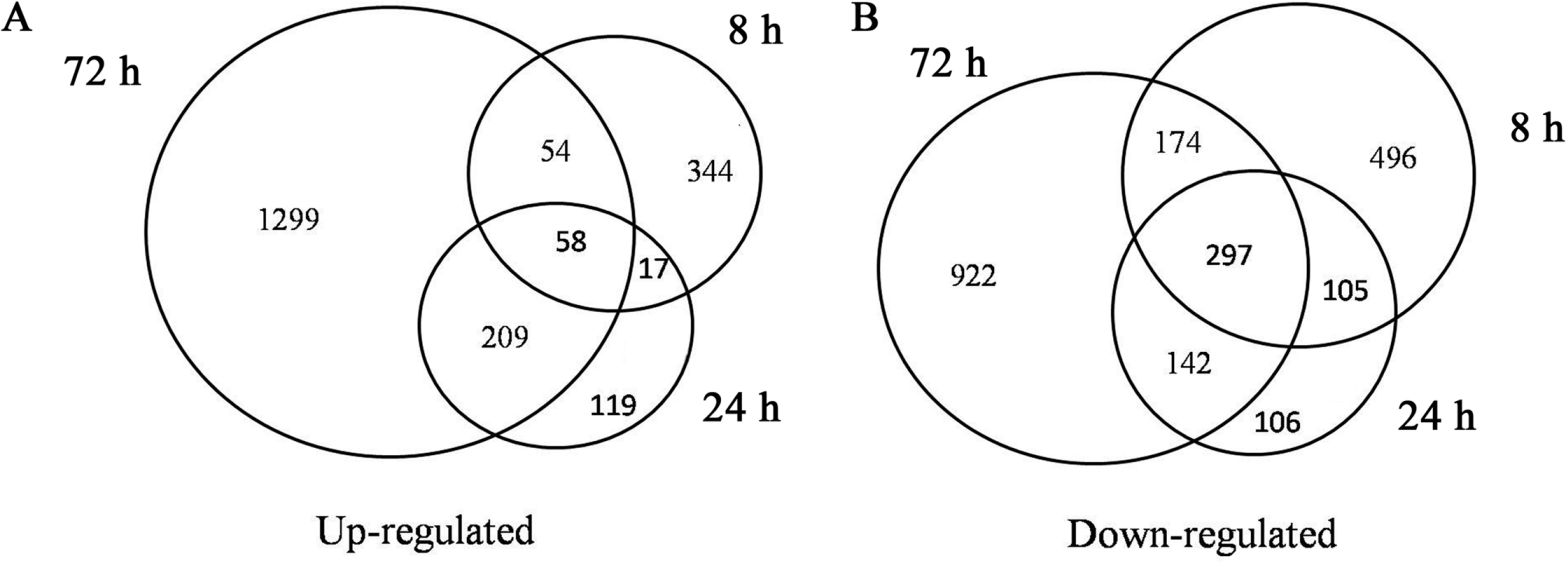
Venn diagrams of sugarcane genes showing changes in the level of transcripts in response to low-K stress. Venn diagrams of up-regulated (**A**) and down-regulated (**B**) genes at different times points after the initiation of low-K treatment. Genes that were up-regulated at at least at one time point (8 h, 24 h and 72 h) were selected for the analysis in (**A**). Genes that were down-regulated at at least one time point (8 h, 24 h and 72 h) were selected for the analysis in (**B**).

A Venn diagram was constructed to investigate the similarities and differences in gene expression changes at different time points. A total of 58 up-regulated genes and 297 down-regulated genes were found at all three time points (Fig 3). A greater percentage of genes were differentially expressed only at 72 h (80.19% up-regulated genes and 60.07% down-regulated genes) compared with 8 h (72.73% and 46.27%) and 24 h (29.53% and 16.38%), whereas 70.47% and 83.69% of the genes that were up- and down-regulated at 24 h also displayed transcriptional changes at 8 h or 72 h (Fig 3).

**Fig 4:**
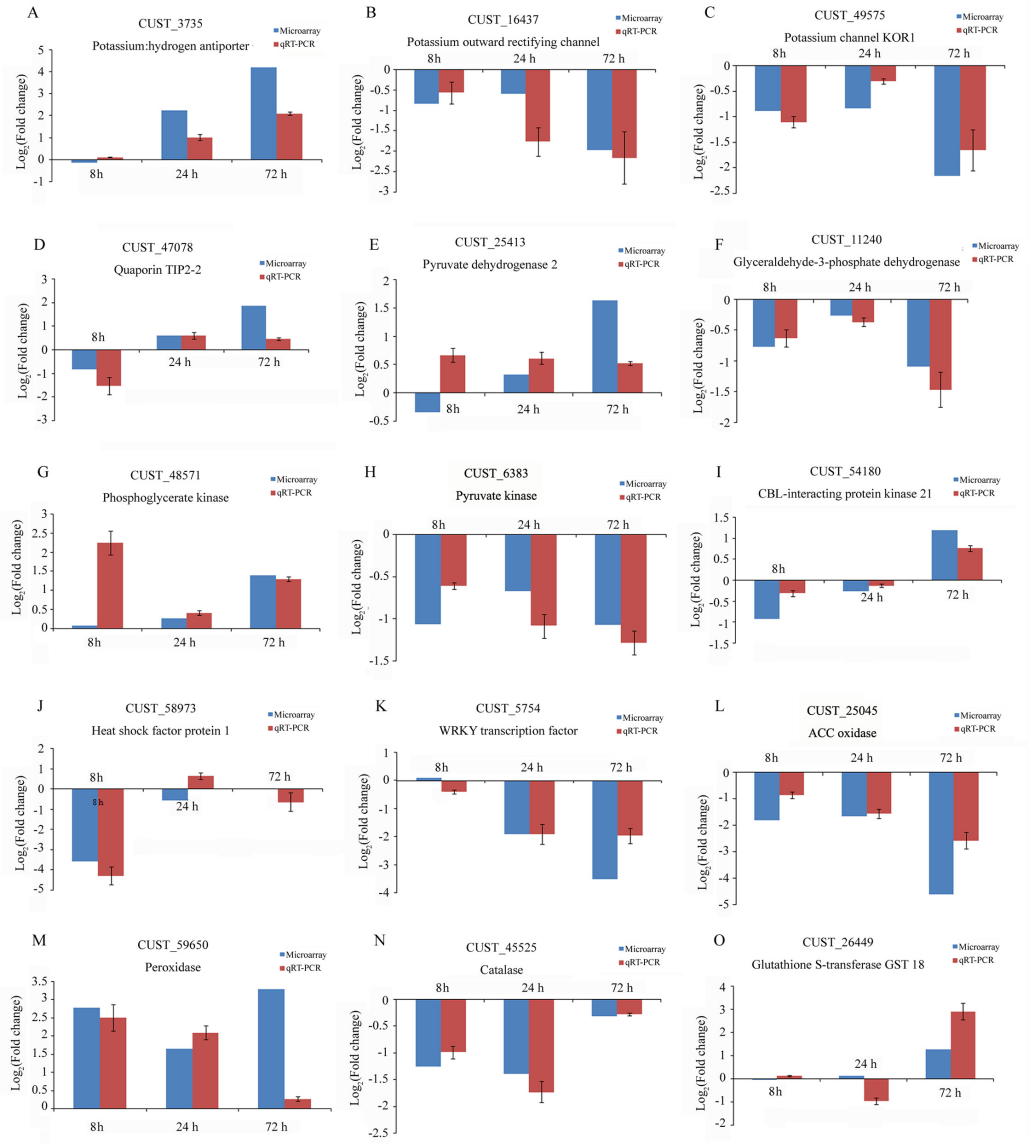
Quantitative real-time PCR confirmation of the transcriptomic profiles of selected genes. The log_2_(LK/CK) values derived from the microarray data of 15 genes were compared with those from qRT-PCR.

To confirm the validity of the microarray data, fifteen differentially expressed genes were selected, including four genes involved in transport, four genes involved in glycolysis and the citrate cycle, three genes involved in calcium signaling and transcriptional regulation, one gene involved in ethylene synthesis and three genes involved in oxidative stress. The expression patterns of these genes were monitored at four time points by qRT-PCR. For most of the fifteen genes, the expression patterns showed somewhat similar trends; however there were slight differences (Fig 4). For example, pyruvate dehydrogenase 2 (CUST_25413) was up-regulated at 72 h based on microarray analysis; however, qRT-PCR analysis revealed that the expression patterns of this gene were similar at the three time points and the level of transcript did not notably increase at 72 h. Conversely, the expression of phosphoglycerate kinase (CUST_48571) did not increase at 8 h based on microarray analysis; however, it was obviously up-regulated based on qRT-PCR. All the expression datasets have been deposited in the Gene Expression Omnibus (GSE61935) at the National Center for Biotechnology Information (http://www.ncbi.nlm.nih.gov/geo/).

### Gene ontology analysis of genes that respond to low-K stress

**Fig 5:**
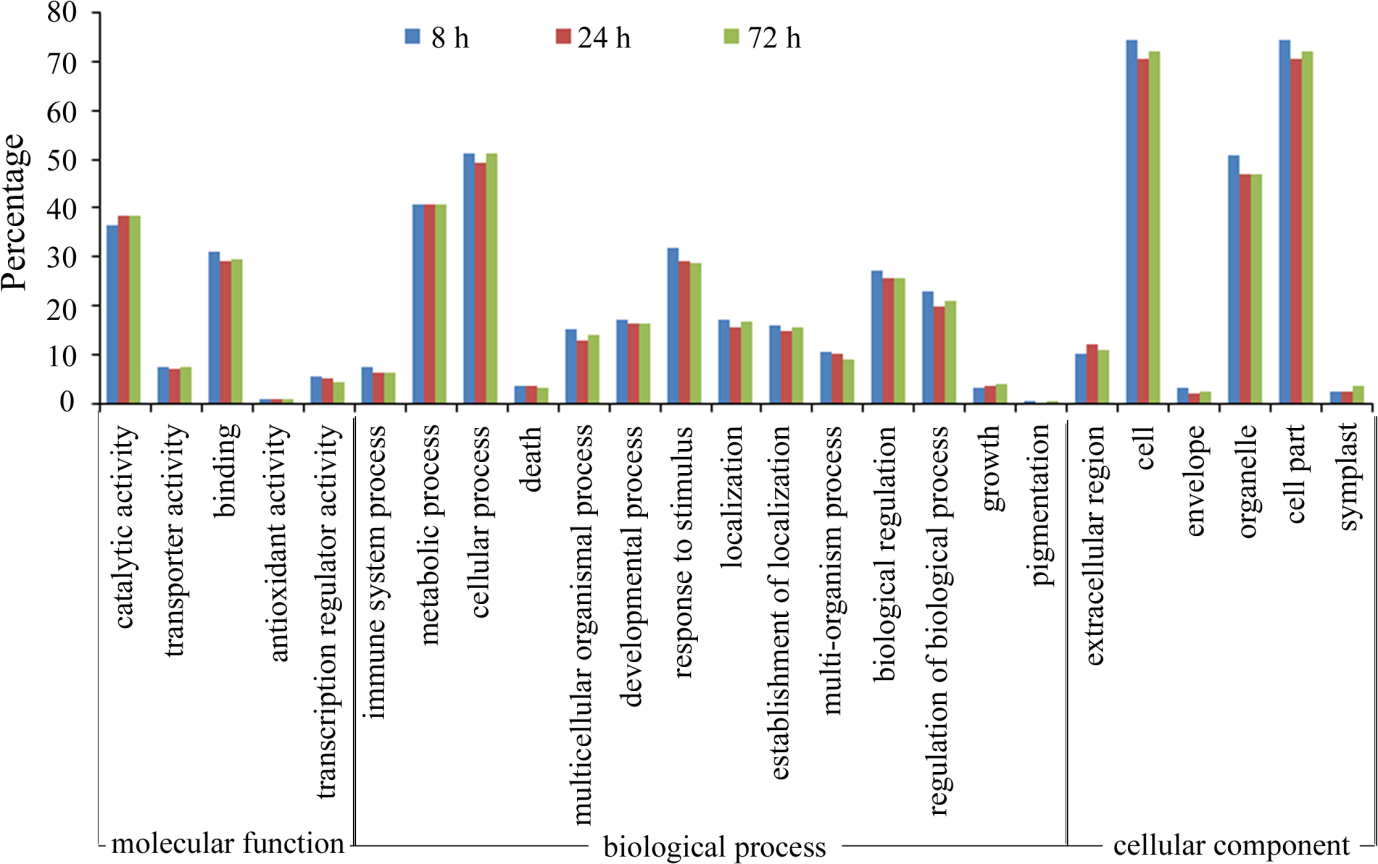
Distribution of the functional GO categories of sugarcane genes that were differentially expressed (P<0.05) after exposure to low-K stress for 8, 24 and 72 h.

Gene Ontology (GO) analysis was used to evaluate the potential function of genes that were differentially expressed under low-K stress. Although the number of low-K responsive genes differed among the three time points, the percentages of genes in the different functional categories were similar (Fig 5). Based on enrichment of genes binned by molecular function, we found that most genes were associated with catalytic activity, binding, transport and transcriptional regulation, accounting for approximately 90% of molecular function (Fig 5). Enrichment analysis of GO terms based on biological processes revealed that the genes involved in metabolic processes, cellular processes, response to stimulus, biological regulation and developmental processes were significantly enriched (Fig 5). In addition, the products of genes that responded to low-K stress were involved in diverse cellular components. Genes in the cell parts category were the most enriched (approximately 70%), followed by organelle genes s (approximately 40%) (Fig 5). However, the percentage of genes in each category was slightly different among the three time points. At 8 h, the percentage of differentially expressed genes in 20 categories (four molecular function categories, 12 biological process categories and four cellular component categories) was higher than that at 24 h or 72 h. The same result was not observed for genes in the other five categories (catalytic activity, growth, pigmentation, symplast and extracellular region).

### Metabolic genes

**Fig 6:**
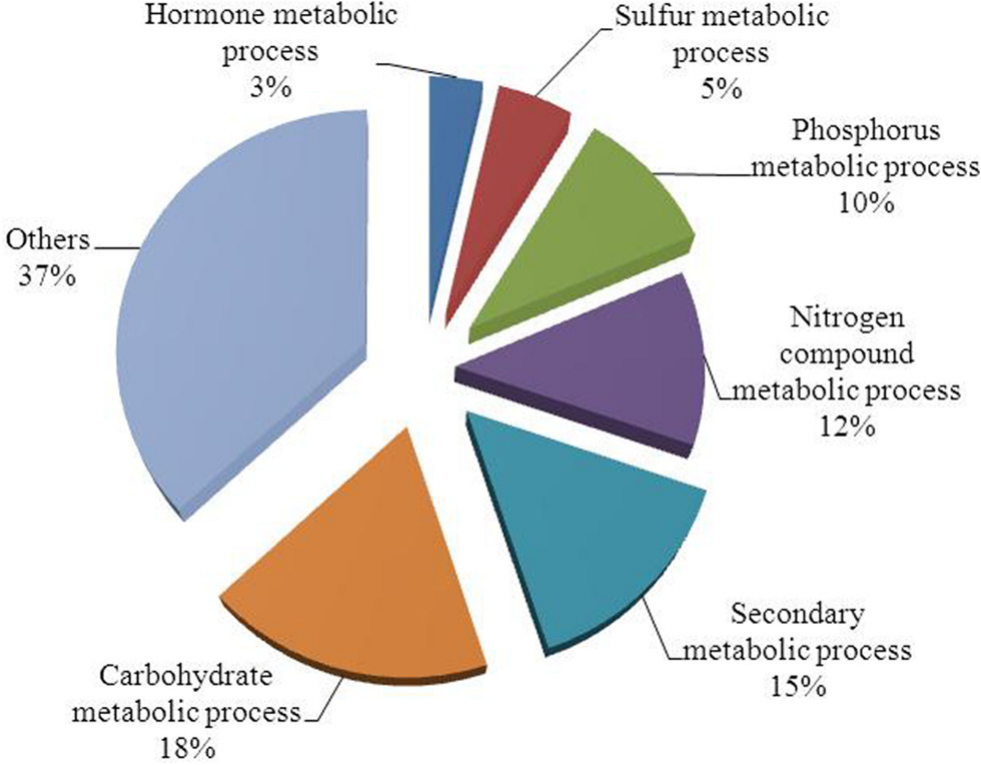
Functional classification of metabolic gene responses to low-K stress.

**Fig 7:**
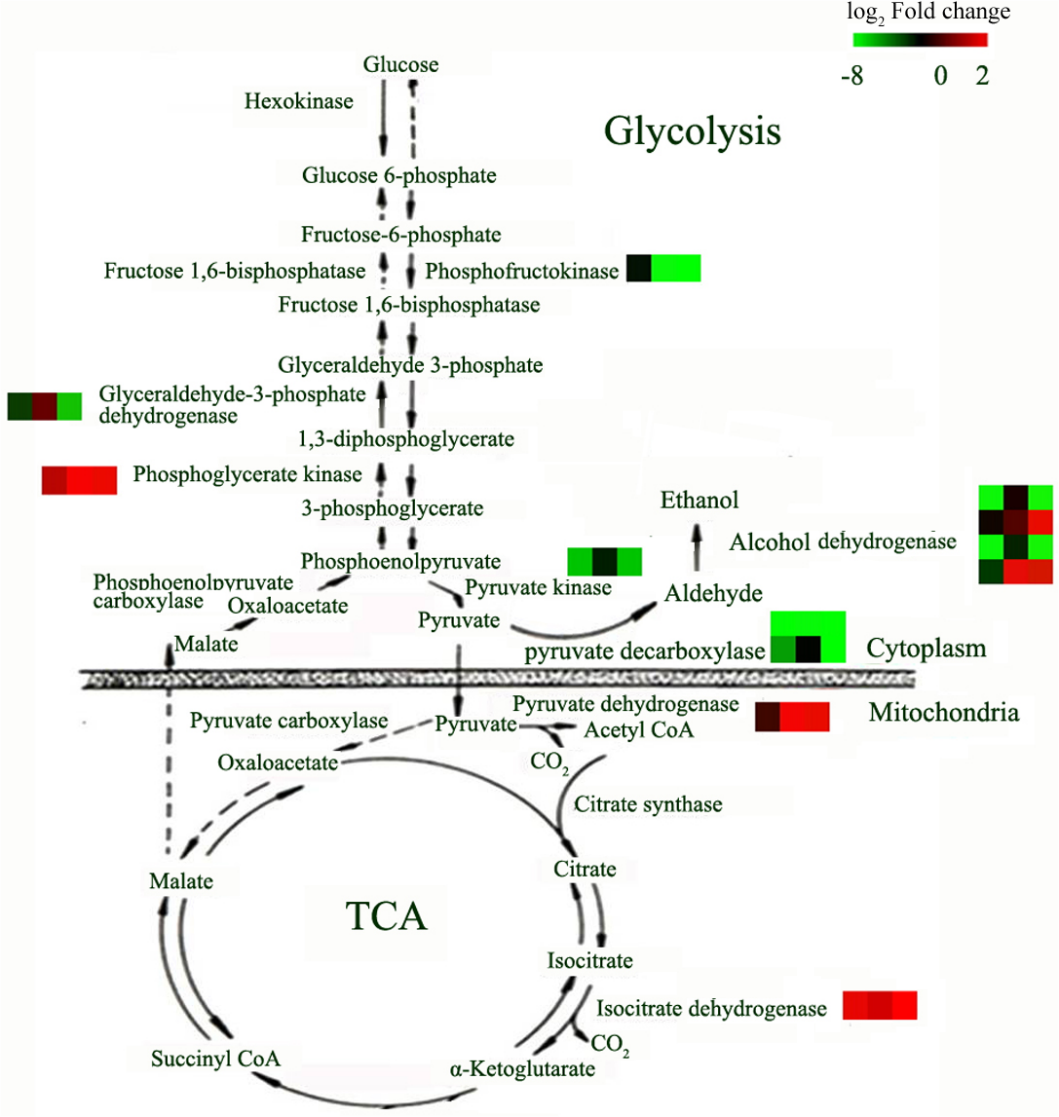
Genes involved in glycolysis and the TCA cycle that displayed altered expression in sugarcane under low-K stress. The relative expression levels at 8 h, 24 h and 72 h are shown adjacent to a color gradient from low (green) to high (red). The probes for the differentially expressed genes were CUST_47734 (phosphofructokinase), CUST_11240 (glyceraldehydes-3-phosphate dehydrogenase), CUST_48571 (phosphoglycerate kinase), CUST_6383 (pyruvate kinase), CUST_15254, CUST_44992 (pyruvate decarboxylase), CUST_5247, CUST_7632, CUST_2997, CUST_45329 (alcohol dehydrogenase), CUST_25413 (pyruvate dehydrogenase), and CUST_53982 (isocitrate dehydrogenase).

It is known that K ions are a cofactor for many metabolic enzymes [27]. Transcriptional changes in genes involved in metabolic processes were detected in this study. GO analysis of differentially expressed genes revealed that approximately 40% of the genes found were involved in metabolic processes. Of these, approximately 12% were related to nitrogen metabolism, 10% to phosphorus metabolism, 18% to carbohydrate metabolism and 15% to secondary metabolism, together accounting for approximately 55% of genes in the metabolic category (Fig 6). We observed that many genes involved in carbohydrate metabolism participated in glycolysis and the citrate (TCA) cycle (Fig 7 and S4 Table). However, the expression patterns of these genes varied. The expression of pyruvate kinase (CUST_6383) rapidly decreased at 8 h, while genes encoding pyruvate decarboxylase (CUST_15254, CUST_44992), 6-phosphofructokinase (CUST_47734), glyceraldehyde-3-phosphate dehydrogenase (CUST_11240), and alcohol dehydrogenase (CUST_5247, CUST_2997) were down-regulated under low-K stress, especially at 72 h. However, the transcription of phosphoglycerate kinase (CUST_48571), pyruvate dehydrogenase (CUST_25413) and NAD-dependent isocitrate dehydrogenase (CUST_53982) increased at 72 h. Therefore, differences in the expression patterns of genes involved in glycolysis and the citrate cycle could disrupt energy production.

Meanwhile, some genes involved in the metabolism of cell wall components, such as cellulose synthase (CUST_40582, CUST_11102, CUST_9367, CUST_14077) and glycosyltransferase (CUST_14014, CUST_45535), were up-regulated at 72 h (S4 Table). In addition, genes involved in the metabolism of soluble acids, such as sucrose synthase (CUST_44128, CUST_42367, CUST_27228) and soluble acid invertase (CUST_59812), were up-regulated at both 24 h and 72 h (S4 Table).

In our datasets, approximately 3% of the differentially expressed genes in the metabolic category participated in hormone metabolic processes. Among these genes, a gene encoding 1-aminocyclopropane-1-carboxylate oxidase (ACC oxidase, CUST_25045) was rapidly down-regulated after 8 h of exposure to low-K stress (S4 Table). A similar result was detected in our qRT-PCR analysis which showed a continuous decrease in the level of transcript as the stress continued (Fig 4).

### Transporters

Absorption and translocation of K in plants occur mainly via by high- and low-affinity K uptake systems, which are mediated by K transporters and channels, respectively [28]. Based on our microarray data, 17 genes related to K transport showed changes in the level of transcripts in response to low-K stress (Table 1). Four K transporters (CUST_1497, CUST_17819, CUST_36971 and CUST_7112) and two K channels (CUST_36270, CUST_41802) were markedly up-regulated after 72 h of exposure to low-K stress. However, four K transporters (CUST_20591, CUST_510, CUST_53063 and CUST_6380) and a K channel (CUST_49258) were rapidly down-regulated after 8 h of exposure to low-K conditions and their expression remained inhibited at 24 h and 72 h. According to cellular component GO analysis, seven out of nine K transporters (CUST_1497, CUST_20591, CUST_36971, CUST_510, CUST_53063, CUST_6380 and CUST_7112) and two out of eight K channels (CUST_27100 and CUST_36270) were likely located in the plasma membrane, while the other eight K transporters/channels might be located in different cellular organelles such as the nucleus, cytoplasm, vacuole, membrane or plasmodesma (S5 Table).

**Table 1:** Genes showing altered expression in sugarcane under low-K stress.

ProbeName	log_2_(LK/CK) ^a^			nr_accession	Description	Function
	8 h vs 0 h	24 h vs 0 h	72 h vs 0 h			
CUST_1497			1.06	XP_003557711	potassium transporter 22-like	potassium transport
CUST_17819			1.20	XP_003576484	potassium transporter 23-like	potassium transport
CUST_20591			-1.25	NP_001060637	potassium transporter 9	potassium transport
CUST_36971			1.42	NP_001048012	Potassium transporter 25	potassium transport
CUST_42207			-1.36	NP_001053859	probable potassium transporter 11	potassium transport
CUST_510	-1.69	-2.06	-1.36	NP_001060637	probable potassium transporter 9	potassium transport
CUST_53063	-1.56			NP_001147472	potassium transporter 10	potassium transport
CUST_6380	-2.12	-1.79	-2.25	AEA08583	high affinity potassium transporter	potassium transport
CUST_7112			1.12	ADR51675	potassium high-affinity transporter	potassium transport
CUST_25267			-1.25	AAX08090	outward-rectifying potassium channel	potassium transport
CUST_16437			-1.94	AAW82753	potassium outward rectifying channel	potassium transport
CUST_27100			-1.06	XP_003626309	sodium/potassium/calcium exchanger	potassium transport
CUST_36270		1.08	1.10	P0C550	potassium channel AKT1	potassium transport
CUST_49258	-1.74	-1.03	-1.56	NP_001105120	potassium channel protein ZMK2	potassium transport
CUST_49575			-2.18	Q653P0	Potassium channel KOR1	potassium transport
CUST_41802			1.08	XP_002868604	potassium channel tetramerization domain-containing protein	potassium transport
CUST_55816	-1.56	-2.00	-1.06	NP_00104741	potassium channel tetramerization domain-containing protein-like	potassium transport
CUST_42158			2.45	ACQ83491	CBL-interacting protein kinase 14	kinases and phosphatase
CUST_51966			1.20	ACQ83503	CBL-interacting protein kinase 03	kinases and phosphatase
CUST_20141	-1.36			ACQ83488	CBL-interacting protein kinase 09	kinases and phosphatase
CUST_13175	-4.06	-2.84	-3.18	ACQ83508	CBL-interacting protein kinase 01	kinases and phosphatase
CUST_7638	-1.64	-1.32		ACQ83502	CBL-interacting protein kinase 30	kinases and phosphatase
CUST_54180			1.18	ACQ83498	CBL-interacting protein kinase 21	kinases and phosphatase
CUST_9371			1.32	ACQ83514	CBL-interacting protein kinase 24	kinases and phosphatase
CUST_49630			1.36	ACQ83509	CBL-interacting protein kinase 15	kinases and phosphatase
CUST_4771	-1.60	1.16	3.36	ACQ83496	CBL-interacting protein kinase 20	kinases and phosphatase
CUST_10946		-1.51	-2.47	ACQ83486	CBL-interacting protein kinase 19	kinases and phosphatase
CUST_28467	1.63			NP_001056378	probable protein phosphatase 2C 51	kinases and phosphatase
CUST_40716	-2.25	-1.84	-3.18	ABB47942	protein phosphatase 2C containing protein	kinases and phosphatase
CUST_36509	-1.25			ABF94415	protein phosphatase 2C containing protein	kinases and phosphatase
CUST_36910	-1.06			NP_001151594	catalytic/ protein phosphatase type 2C	kinases and phosphatase
CUST_60543			-1.18	NP_001148728	protein phosphatase 2C	kinases and phosphatase
CUST_55757			1.41	ACQ83549	calcineurin B-Like protein 04	calcineurin B-like protein
CUST_25359	-1.29			ACQ83548	calcineurin B-Like protein 07	calcineurin B-like protein
CUST_2176	-2.40	-1.29	-2.84	ACF95746	MYB transcription factor MYBAS1	transcription regulators
CUST_27453	-2.56	-1.84	-2.84	P20025	Myb-related protein Zm38	transcription regulators
CUST_28701			-1.89	ACG36390	MYB59	transcription regulators
CUST_61488	-1.56		-2.40	EMT32647	Myb-related protein MYBAS2	transcription regulators
CUST_58112	-1.22	-1.84	-2.06	AFO85372	NAC1, partial	transcription regulators
CUST_470	-1.51	-1.56	-1.47	AAW62955	NAC23	transcription regulators
CUST_16274	-3.06	-1.89	-2.47	DAA37711	putative NAC domain transcription factor	transcription regulators
CUST_45563	-2.40	-2.25	-2.74	DAA46404	putative NAC domain transcription factor	transcription regulators
CUST_48354		-1.18		DAA55107	putative NAC domain transcription factor	transcription regulators
CUST_54637	1.93			DAA46243	putative AP2/EREBP transcription factor	transcription regulators
CUST_12974	-1.89	-2.32		AFW63369	putative AP2/EREBP transcription factor	transcription regulators
CUST_13792			-1.06	CAM35490	ethylene responsive transcription factor	transcription regulators
CUST_20821	-1.69		-2.12	NP_001147529	ethylene-responsive element binding protein	transcription regulators
CUST_46633	-2.40		-2.84	NP_001146913	ethylene-responsive factor-like protein	transcription regulators
CUST_58973	-3.64			NP_001152657	heat shock factor protein 1	transcription regulators
CUST_41064	-2.94	-1.89	-2.32	ACM42161	heat shock protein 70.58	stress-related
CUST_22888			1.36	AFV66576	heat shock protein 70,	stress-related
CUST_60607	1.97			AFK73383	small heat-shock protein	stress-related
CUST_50618			1.19	CAA78738	heat shock protein hsp82	stress-related
CUST_7987		-1.23	-1.21	DAA41888	class IV heat shock protein	stress-related
CUST_57095			1.51	NP_001104981	glutathione S-transferase12	oxidative stress-related
CUST_61600			1.42	DAA43222	TPA: glutathione S-transferase GST 11	oxidative stress-related
CUST_26449			1.28	NP_001104984	glutathione S-transferase GST 18	oxidative stress-related
CUST_27585		-1.56		AAL73498	lipoxygenase	oxidative stress-related
CUST_27568	1.29			ACG32380	superoxide dismutase	oxidative stress-related
CUST_45525	-1.25	-1.40		ABQ44283	catalase	oxidative stress-related
CUST_26339	-1.06			YP_006316948	glutathione peroxidase	oxidative stress-related
CUST_43430			1.37	AFW87396	peroxiredoxin-5	oxidative stress-related
CUST_56046		1.36	1.70	NP_001106040	peroxidase 70 precursor	oxidative stress-related
CUST_45804			2.07	DAA45509	plant peroxidase family protein	oxidative stress-related

Note: ^a^ The fold change of each gene was transformed into “log_2_”. The gene with log_2_(LK/CK) ≥ 1 were defined as up-regulated; The genes with log_2_(LK/CK) ≤ -1 were defined as down-regulated.

In addition to K transporter genes, we found that the level of transcripts of some genes involved in water transport changed in sugarcane roots during K deficiency. According to our microarray data, four aquaporin genes (CUST_56031, CUST_44135, CUST_52950 and CUST_47078) were rapidly down-regulated after 8 h of K starvation. However, the expression levels of these genes recovered after 24 h and subsequently increased as the stress continued (S5 Table).

### Kinases and phosphatases

Protein kinases and phosphatases participate in a large number of distinct perception and signaling pathways that play important roles in cellular development. Based on our microarray data, a total of 405 genes encoding kinases and phosphatases were found to be transcriptionally altered by K starvation (S6 Table), accounting for approximately 10% of the low-K-responsive genes and suggesting that phosphorylation and dephosphorylation may be very important regulatory mechanisms for the sugarcane response to K deficiency. Of these genes, 11 CIPKs and 5 type 2C protein phosphatases were found to be differentially expressed in response to low-K stress (Table 1). Six CIPK genes (CUST_42158, CUST_51966, CUST_54180, CUST_9371, CUST_49630 and CUST_4771) were markedly up-regulated after 72 h of exposure to low-K conditions, and a protein phosphatase 2C (CUST_28467) was up-regulated after 8 h of low-K stress (Table 1). Increased expression of these kinase and phosphatase genes may regulate K uptake and K homeostasis in sugarcane during K deficiency. We also found that two members of the CBL family (CUST_55757, CUST_25359) were differentially expressed. CUST_55757 was up-regulated at 72 h, while CUST_25359 was down-regulated at 8 h (Table 1). There were also many other types of kinases that were differentially expressed in our microarray data, including an S-receptor-like serine/threonine-protein kinase (CUST_422), three MAPKKK family protein kinases (CUST_4402, CUST_29659 and CUST_49837), and three wall-associated receptor protein kinases (CUST_44285, CUST_85 and CUST_19991) (S6 Table). These kinases might participate in the regulation of the sugarcane response to low-K stress and form a complicated biological regulation network.

### Transcriptional regulation

Our microarray results showed that 191 genes encoding transcriptional regulators were differentially expressed during low-K stress, including MYB (4), NAC (5), AP2-EREBP (2), WRKY (1), ERF (3), bHLH (2), TGA6 (1), E2F3 (1), RCBF3 (1) and HSF (1) family members (Table 1). The other genes were defined as putative transcription factors (S7 Table). Among the genes we identified, five transcription factors involved in ethylene signaling (CUST_54637, CUST_12974, CUST_13792, CUST_20821, and CUST_46633) were differentially expressed (Table 1). The expression of three ethylene-responsive transcription factors (CUST_12974, CUST_20821 andCUST_46633) was down-regulated in response to low-K stress within 8 h, and one (CUST_13792) showed marked down-regulation only at the 72 h time point. However, an AP2/ERF transcription factor (CUST_54637) was up-regulated in response to low-K stress at 8 h. In addition, four putative Myb family transcription factors (CUST_2176, CUST_27453, CUST_28701 and CUST_61488) and five NAC family transcription factors (CUST_16274, CUST_45563, CUST_48354, CUST_58112 and CUST_470) were found to be down-regulated at at least one of the three time points (8 h, 24 h, and 72 h) (Table 1). We also noticed that a heat shock factor protein (CUST_58973) was markedly down-regulated at 8 h. However, the heat shock proteins regulated by heat shock factor showed differential expression patterns in our datasets. Three heat shock proteins (CUST_22888 CUST_50618 and CUST_60607) were markedly up-regulated at 8 h or 72 h, whereas two members (CUST_41064 and CUST_7987) were down-regulated (Table 1).

### Oxidative stress-related genes

Reactive oxygen species (ROS) play crucial roles in plant stress responses, development, response to pathogens, and many other physiological processes [29]. However, the excessive production of ROS will cause oxidative stress. In our study, a total of 44 oxidative stress-related genes were differentially expressed under low-K stress conditions (S8 Table and Table 1). At 8 h, 24 h and 72 h, the number of up-regulated genes was 3.75-, 1.75- and 3-fold greater than the number of down-regulated genes, respectively. Among these genes, a superoxide dismutase (CUST_26449) was rapidly up-regulated at 8 h of exposure to low-K conditions. Three glutathione S-transferases (CUST_57095, CUST_61600 and CUST_26449), a peroxiredoxin gene (CUST_43430) and two peroxidases (CUST_56046, CUST_45804) were up-regulated at 72 h. However, a catalase (CUST_45525) and a glutathione peroxidase (CUST_26339) were rapidly down-regulated at 8 h under low-K stress, while the expression of a lipoxygenase (CUST_27585) decreased at 24 h (Table 1).

## Discussion

Potassium is an essential macronutrient that is crucial for plant growth and development. Previous work has demonstrated that plants respond rapidly to K deficiency. For example, hyperpolarization of the cell membrane potential occurs within a few minutes of a decrease in extracellular K, an event that represents the earliest detected event in the plant response to K deficiency and may act as one of the sensing-related signals for downstream responses [30]. After hyperpolarization, a series of biochemical and physiological reactions occur in plant cells that include both short- and long-term responses [31]. Transcriptional profiling of rice genes has revealed that many genes are differentially regulated 6 h after the start of low-K stress, and the number of differentially expressed genes increases with time [14]. Our work examining the relationship between the K content of sugarcane roots and changes in the level of transcripts also demonstrated a rapid response occurring after only 8 h of exposure to low-K stress. The K content of sugarcane roots continued to decrease after this time point, and the number of differentially expressed genes increased at 72 h (Fig 1 and Fig 3), which was consistent with the results of a transcriptional profiling study of rice genes under low-K stress [14]. However, many genes showed differential expression only at 72 h, and most of the genes that were differentially expressed at 24 h were also detected at 8 h or 72 h (Fig 2), suggesting that short-term responses to low-K stress might differ from long-term responses and that 24 h might be an important time point for initiating the long-term response in sugarcane. GO analysis of differentially expressed genes revealed that as the stress continued, higher percentages of genes in the catalytic activity and growth categories were detected especially at 72 h (Fig 5). These results indicated that more genes downstream of metabolic and regulatory networks might be activated under long-term stress, which would likely eventually affect sugarcane growth.

As cofactor for many enzymes, K ions participate in many metabolic processes [27]. K-activated enzymes are thought to act as K sensors in the cytoplasm [28]. Transcriptional profiling studies of genes under low-K stress in rice, soybean and *Arabidopsis* have revealed that genes involved in metabolic processes account for a higher percentage of genes relative to the total number of stress-response genes, and these studies have detected expression changes in many crucial enzymes that are crucial for metabolism, such as enzymes involved in N, S, and P assimilation, pyruvate synthesis and sugar metabolism [14,16,17]. These changes indicate that transcriptional regulation of metabolic enzymes may be very important for plants when adapting to K deficiency [32]. In this study, nearly half of the genes that were responsive to low-K stress were involved in metabolic processes, including genes related to C, N, P and secondary metabolism (Fig 5). Among the metabolic enzymes, pyruvate kinase acts as a central regulator of C/N metabolism [33]. The activity of pyruvate kinase can be directly inhibited after long-term K-deficiency, which induces a significant reduction in the cytoplasmic pyruvate content of root cells and leads to inhibition of glycolysis and many downstream metabolic processes (such as the TCA and GS/GOGAT/GDH cycles) [32]. Here, we detected the down-regulation of pyruvate kinase (CUST_6383) after short-term (8 h) low-K stress (S4 Table and Fig 7). This result suggested that the inhibition of pyruvate kinase occurs not only on the enzymatic level but also on the transcriptional level. The rapid inhibition of pyruvate kinase under low-K stress might be one of the earliest responses to K deficiency in sugarcane. Meanwhile, we also found several genes other than pyruvate kinase that are involved in glycolysis and the citrate (TCA) cycle differentially expressed in response to low-K stress. Changes in the levels of transcripts of genes in the glycolytic pathway such as pyruvate kinase, glyceraldehyde 3-phosphate dehydrogenase and short-chain alcohol dehydrogenase, were detected in *Arabidopsis* under low-P and low-N conditions [34,35], suggesting that transcriptional regulation of carbohydrate metabolism enzymes, especially those involved in glycolysis and the TCA cycle, might be essential for plant survival under low nutrient conditions.

In plants, K transporters and channels function in the absorption and translocation of K [28]. To date, many plant genes encoding K transporters and channels have been cloned, including *AtAKT1*, *TaAKT1*, *AtHAK5*, *HvHAK1*, and *OsHAK1*, and have been found to mediate the uptake of K under low-K conditions [7,9,10,36,37]. In our microarray experiment, we detected 17 genes related to K transport that were differentially expressed in response to low-K stress (Table 1 and S5 Table). According to cellular component GO analysis, the products of these genes were located in different cellular organelles (S5 Table), which suggested that low-K stress might not only affect the uptake of K but also the transport of K in cellular organelles. However, these genes showed differential expression changes, especially K transporters/channels located in the plasma membrane. The differential changes in the levels of transcripts involved in K transport might be a strategy employed by the plant to cope with K deficiency. The up-regulation of three K transporters (CUST_1497, CUST_36971 and CUST_7112) and a K channel AKT1 (CUST_36270) in the plasma membrane might be involved in K uptake in sugarcane under low-K conditions.

There are two primary signaling pathways that play important roles in regulating K uptake. The first well-known pathway is the Ca^2+^ signaling pathway, which is mediated by CBL-CIPK. Plant-specific serine/threonine protein kinases, or CIPK proteins, can exclusively interact with calcineurin B-Like protein and form a CBL-CIPK complex, which in turn constitutes a specific regulatory network of Ca^2+^ signaling in plant cells [38]. In plants, the CBL-CIPK complex (CBL1-CIPK23, CBL9-CIPK23, CBL4-CIPK6, CBL3-CIPK9) functions in response to low-K stress and is essential for regulating K uptake [11,39,40]. Additional work has revealed that phosphatase PP2CA also participates in the regulation of K uptake via K channels [41]. In sugarcane, two CBL genes, as well as many genes in the CIPK and PP2C families, were differentially expressed in response to low-K stress (Table 1), indicating that a CBL-CIPK-PP2CA pathway may also be involved in regulating K uptake under low-K stress.

The other signaling pathway responsible for low-K tolerance is mediated by ethylene. Ethylene content is significantly increased after only 6 h of K deficiency, and the transcription of genes involved in ethylene biosynthesis also increase in response to these conditions [42]. Under low-K stress, ethylene stimulates the production of ROS [12]. Then, ROS regulate the expression of *AtHAK5* and stimulate root hair elongation, which enhances K uptake and stress tolerance [43]. Genes involved in the ethylene synthesis pathway, such as ACC synthase were identified in a study on comparative transcriptome profiling of wild barley genes responsive to low-K [15]. However, in our study, we found that an important gene in the ethylene synthesis pathway, ACC oxidase, was down-regulated at three time points (S4 Table and Fig 4). ACC oxidase functions to catalyze the conversion of 1-aminocyclopropane-1-carboxylic acid to ethylene, which is one of the most important steps in ethylene synthesis in plants. The down-regulation of ACC oxidase genes disrupts ethylene synthesis in sugarcane under conditions of low-K stress.

Commonly, transcription factors regulate the expression of stress response genes and help plants to overcome biotic and abiotic stress [44–46]. The differential expression of transcription factors, including many stress-related transcription factors, such as Myb, NAC, WRKY family transcription factors has been detected in rice, wild barley, soybean and *Arabidopsis* [14–17]. The AP2/ERF transcription factor RAP2.11 is an important component of the low-K signaling pathway. This transcription factor can be induced by ethylene and ROS and directly binds to the promoter of *AtHAK5* to regulate its expression in response to low-K stress [13]. However, most of the ethylene-responsive transcription factors in our datasets, including AP2/ERF family members, were rapidly down-regulated in response to low-K stress (Table 1). The decrease in the expression of genes involved in both ethylene synthesis and the ethylene signaling pathway indicated that the function of ethylene in regulating low-K responses in sugarcane might be different from that in *Arabidopsis*.

ROS signals are commonly induced under nutrient deprivation conditions, including deficiencies in K, N, P and S [28,47]. ROS is an important signaling component; thus, its production and elimination are important for plant cells. In plants, peroxidase, cytochrome P450, and glutathione S-transferase participate in the production and elimination of ROS [48]. In our datasets, a number of genes involved in oxidative stress were differentially expressed, including lipoxygenase, glutathione S-transferase, superoxide dismutase and peroxidase. Meanwhile, a high percentage of genes involved in oxidative stress were up-regulated at the three time points, especially at 72 h (S8 Table). Up-regulation of genes in oxidative stress-related categories would help to eliminate the excessive ROS and maintain the balance of ROS in sugarcane, which might be a mechanism by which sugarcane can cope with low-K stress.

## Conclusions

In the present study, we performed a microarray-based comparative investigation to assess expression changes in sugarcane genes in response to low-K stress. The microarray data showed that a total of 4153 genes were differentially expressed under low-K conditions and most of the changes appeared within 72 h. GO enrichment analysis revealed that genes involved in metabolic processes, cation binding, biological regulation, transport, and transcriptional regulation were enriched. Genes involved in the Ca^+^ signaling and ethylene pathways showed changes in the level of transcripts in response to low-K conditions, suggesting that they may play important roles in sugarcane responses to K deficiency. However, there were also many ambiguous tags and unannotated genes we were unable to explore in the current study. Future increases our knowledge regarding the sugarcane genome may resolve some of these ambiguities and lead to the discovery of large numbers of genes responsible for low-K tolerance. Although the experiments in which we profiled genes responsive to low-K stress were not replicated in the current study, these results will provide valuable clues for further studies. Further verification of these differentially expressed genes via transgenic technology could lead to the improvement of sugarcane resistance to K deficiency.

## Supporting Information

S1 Table Sequences of probes used for the custom sugarcane genome array. (XLSX)

S2 Table Primers used for qRT-PCR analysis. (XLSX)

S3 Table Numbers of differentially expressed genes in sugarcane under low-K stress. (XLSX)

S4 Table Genes involved in metabolic processes showing altered expression in sugarcane under low-K stress. (XLSX)

S5 Table Transporter-encoding genes showing altered expression in sugarcane under low-K stress. (XLSX)

S6 Table Genes encoding kinases and phosphatases showing altered expression in sugarcane under low-K stress. (XLSX)

S7 Table Genes involved in transcriptional regulation showing altered expression in sugarcane under low-K stress. (XLSX)

S8 Table Genes involved in oxidative stress responses showing altered expression in sugarcane under low-K stress. (XLSX)

S9 Table Genes involved in processes not listed above that are differentially expressed under low-K stress. (XLSX)
